# The Louisiana Medical Society Versus the Editorial Staff of the New Orleans Medical and Surgical Journal

**Published:** 1888-08

**Authors:** Joseph Jones


### LOUISIANA STATE MEDICAL SOCIETY VERSUS THE EDITORIAL STAFF OF THE NEW ORLEANS MEDICAL AND SURGICAL JOURNAL.

*To the Members of the Louisiana State Medical Society*:

GENTLEMEN—The undersigned respectfully direct the attention of the officers and members of the Louisiana State Medical Society to a matter of importance, which was brought to our notice on the 11th of June, 1888, by our most venerable and active members.

The eminent physician and surgeon asks: "Have you read in the June issue of the *New Orleans Medical and Surgical Journal*, the editorial strictures upon the last meeting of the Louisiana State Medical Society ?"

"How shall such ill-natured, scurrilous criticisms be met ?"

"What is demanded of the physicians attending the meeting at Monroe, and how shall they meet the rude, unjust and unprofessional attack ?"

The *ill-natured*, *scurrilous*, *unjust* and *unprofessional criticisms* alluded to are to be found in the June number of the *New Orleans Medical Journal*, 1888, pp. 984–987, under the head, "LEADING ARTICLES," "THE TENTH ANNUAL MEETING OF THE STATE MEDICAL SOCIETY."

The quotations from the "Editorial" will be confined to such portions as will illustrate the *animus* of the editorial staff of the *New Orleans Medical and Surgical Journal*, in its attempt to defame and destroy the Louisiana State Medical Society.

The editors of the *New Orleans Medical and Surgical Journal* say: "Although, as we stated in our last number, owing to duties from which there was no escape, none of our staff was able to be present at the annual meeting of the State Medical Society in Monroe, we gather from the official minutes of the Recording Secretary, which we published, that the meeting was characterized by the same indifference, idleness and slip-shod irresponsibility that has for years made the Medical Society of Louisiana a disgrace instead of a pride to the profession of the State." pp. 984.

A meeting of the Medical Society of any one of our sister States means the assemblage of all the best and wisest members of the profession to consider well-digested plans for the advancement of our interests and for the regulation of our conduct ; to hear and to discuss the latest and most valuable contributions that they are able to offer for mutual help and profit.

"*A meeting of the Louisiana Medical Society is the straggling together in some locality of a dozen or so of languid, inconsequent, unprepared medical men, bent for the most part upon a few days of rest, cigar-smoking and story-telling.*" . . . p. 985.

"It is in no captious spirit that we write these lines, but it must have become apparent to every earnest man that the Louisiana State Medical Society should either undergo a great awakening and revivication, or be abandoned, cleared away as a useless time-consumer, and cumberer of the earth." p. 987.

It is evident from the preceding quotations that the editorial staff of the *New Orleans Medical and Surgical Journal* have, without provocation and without warning, attacked not merely the body of earnest laborers, patriotic and skilled physicians, who assembled at Monroe on the 25th of April, 1888, but have endeavored, through the pages of the *Journal*, to arraign the intelligence and medical attainments of the entire body of medical gentlemen composing the Louisiana State Medical Society. No honorable member of the medical profession objects to honest and courteous criticism, designed to advance the cause of science; but the wholesale abuse and slander of a medical society, comprised of two hundred gentlemen, distinguished for their professional attainments and benevolent labors, for the advancement of the highest and best interests of the citizens of Louisiana, should receive such brief but decided condemnation as will place the authors in their true light before the medical profession. We will consider this subject under two aspects, namely:

First. The relations of the editorial staff of the *New Orleans Medical and Surgical Journal* to the Louisiana State Medical Society.

Second. The truth or falsehood of the statement of the staff of the *New Orleans Medical and Surgical Journal* with reference to the meetings of the Louisiana State Medical Society in Monroe and elsewhere.

### 1ST. THE RELATIONS OF THE EDITORIAL STAFF OF THE NEW ORLEANS MEDICAL AND SURGICAL JOURNAL TO THE LOUISIANA STATE MEDICAL SOCIETY.

The following table has been drawn up from the records of the Louisiana State Medical Society, and shows that of the eight editors, seven are members of the Louisiana Medical Society, and five of this number have received appointments as members and chairmen of standing committees, and two of them have been honored with the position of Vice-President, and one of them holds the position of Treasurer and Librarian.

EDITORIAL STAFF NEW ORLEANS MEDICAL AND SURGICAL JOURNAL.

| NAME. | ADDRESS. | Date of Graduation, Medicine. | Date of joining La. State Med. Soc'ty. | Honors and Positions Conferred by the Louisiana State Medical Society and Dates thereof. |
|---|---|---|---|---|
| Geoge B. Lawrason, M. D. | New Orleans | 1883 | 1884 | Member of Committee on Publication. |
| Henry Dickson Bruns, M. D. | New Orleans | 1881 | 1884 | Annual Orator, address delivered at New Iberia, 1886; Chairman of Committee on Revision of Constitution and By laws, 1886, 1887; Member of Committee on Scientific Essays, 1887, 1888; Committee on Organization, 1887, 1888; on Necrology, 1887, 1888; Vice-President Second Congressional District of Louisiana, 1887, 1888. |
| F. W. Parham, M. D. | New Orleans | 1879 | 1879 | Treasurer and Librarian, 1885, 1888; Member of Committee on Publication, 1886, 1888; Committee on Revision of Constitution, 1886-7; Committee on Organization, 1887-8; Committee on Necrology, 1887, 1888; Vice-President First Congressional District, 1887, 1888. |
| John H Bemis, M. D. | New Orleans | 1878 | 1884 | |
| P. E. Archenard, M. D. | New Orleans | 1882 | 1885 | |
| A. McShane, M. D. | New Orleans | 1882 | 1885 | |
| L. Bemis, Esq. | New Orleans | ......... | ......... | |
| H. W. Blanc | New Orleans | 1884 | 1887 | |

From the preceding record it is evident that of the editorial staff of the *New Orleans Medical and Surgical Journal* not one has served in the ranks of the profession more than ten years; the stench, therefore, which this editorial of June, 1888, has created in the nostrils of many of the venerable and experienced members of the Louisiana State Medical Society must be referred to the inexperience and enthusiastic conceit of youth. At the time of the meeting of the State Medical Society at Monroe, Dr. F. W. Parham held the position of Treasurer and Librarian and Vice-President of the First Congressional District. Dr. H. D. Bruns held the position of Vice-President of the Second Congressional District, Acting Chairman of the Committee on Scientific Essays, Chairman of the Committee of the Revision of the Constitution, and member of the Committee on Organization and Necrology. Dr. Archenard was a member of the Committee on Publication.

The editorial staff of the *New Orleans Medical and Surgical Journal* have been honored and trusted by the Louisiana State Medical Society, and they have, when it suited their convenience, used the valuable reports and original papers read and presented by the officers and members of the society to fill up the pages of their journal. The most important matter for the action of the society at its tenth annual session was the discussion, adoption or rejection of the revised constitution and by-laws, which had been printed in parallel columns with the old constitution in the transactions of 1887.

Dr. Bruns was the originator of this movement at the New Iberia meeting, and the committee consisted of Dr. H. D. Bruns, Dr. F. W. Parham and Dr. A. B. Miles. Dr. Bruns was not only absent from the meeting at Monroe, but, as far as our information extends, failed to address an official communication to the Louisiana State Medieal Society explaining his failure to appear.

Third. Whatever may be the limits of the circulation or professional standing of a medical journal, it should be borne in mind that it is an integral portion of the medical press and of the country, and through its exchanges reaches far and wide for good or for evil.

If the "*Medical Society of Louisiana has been for years a disgrace instead of a pride to the profession of the State,*" why did the editors of the *New Orleans Medical and Surgical Journal* hold membership, enjoy its honors and use its valuable papers to enlighten and benefit their subscribers?

If the condition of things described by the editorial staff of the *New Orleans Medical and Surgical Journal* really existed, why did not these editors have the manhood to denounce these evils in person, face to face with their fellow-members, and from the high positions to which they had been elected? Was it just and right to stab their fellow-members and friends without warning? Was it just and right to use the medical press to abuse and traduce their fellow-members?

Does the holding of position in the great army of American medical editors absolve men from the ordinary rules and dictates of friendship and professional courtesy?

2D. THE TENTH ANNUAL MEETING OF THE LOUISIANA STATE MEDICAL SOCIETY AT MONROE, LOUISIANA, APRIL 25, 1888.

The gentlemen comprising this assemblage were about equal in numbers to those assembled at New Iberia and Alexandria; the number of new members elected being 12 at New Iberia, 16 at Alexandria and 16 at Monroe; total new members added to the Louisiana State Medical Society in 1886, 1887 and 1888, 44. The total number of permanent members previous to the meeting at New Iberia was 150; thus we have during the past three years an increment of nearly 30 per cent., or more accurately 29.3 per cent. When we reflect that the Louisiana State Medical Society had practically no existence until 1878, we must regard the steady increase in its numbers as proof that the medical profession of Louisiana regard its membership as an honor.

In medical attainments, earnestness, courteous and manly bearing, the medical gentlemen who assembled at Monroe were the peers of the members of any similar gathering in Louisiana or any other State in this Republic. The daily sessions were distinguished by careful and continuous scientific and executive labors, by harmony, and by a courteous regard to parliamentary rule. The entire time of the session was devoted to the reading and discussion of papers and the transaction of important business. The relative amount of scientific work accomplished may be seen from the following record of the past three meetings of the State Medical Society: Number of papers read at the New Iberia meeting in 1886, eleven; number of papers read at Alexandria, 1887, sixteen; number of papers read at Monroe, 14; total 41.

The character of the labors of the State Medical Society, at Monroe, will be shown to the medical profession in due time in the printed transactions of 1888, which will vindicate the high attainments of the representatives of the regular medical profession.

The results of the meeting at Monroe are especially worthy of note, when it is considered that the State of Louisiana had just passed through a heated political campaign which had engaged the close attention and monopolized the energies of the citizens for several months; and when it is also remembered that Monroe is the most distant town from the large centres of population at which a meeting has been held, and is also comparatively inaccessible.

Louisiana suffered severely during the civil war of 1861–1865, which overthrew her system of labor, destroyed her levees, and fastened upon her vitals a horde of rapacious, unprincipled and unscrupulous, insatiable vultures.

It was not until 1877 that there was any proper recognition by the Federal Government of the political and civil rights of the citizens of Louisiana.

With this change came the revival of the State Medical Society, and the little band of medical men devoted to the interests of the citizens and profession of their beloved State has been steadily marching onward with the hope that they will in time enroll within their ranks every honorable member of the regular medical profession, and gradually perfect and establish all measures relating to the elevation and protection of the medical profession of Louisiana.

IT IS NOT TRUE, THEREFORE, that "the tenth annual meeting of the State Medical Society at Monroe, Louisiana, was characterized by indifference, idleness and slip-shod irresponsibility."

It is not true, therefore, that "the meetings of the Louisiana Medical Society are the straggling together of a dozen or so of languid, inconsequent, unprepared medical men, bent for the most part upon a few days of rest, cigar-smoking and story-telling."

Respectfully,

JOSEPH JONES, M. D.,

Ex-President Louisiana State Medical Society, presiding officer at Monroe, Louisiana, April 25 and 26, New Orleans.

R. H. Day, M. D., Louisiana, ex-President Louisiana State Medical Society, Baton Rouge, La.

J. W. Dupree, M. D., ex-President Louisiana State Medical Society, Baton Rouge, La.

G. G. Belford, M. D., Bastrop, La.

F. M. Thornhill, M. D., ex-Vice-President Fifth Congressional District, Arcadia, La.

J. G. Bridges, M. D.

J. M. Patterson, M. D., Arcadia, La.

P. R. Fox, M. D., ex-President Louisiana State Medical Society, Jesuit's Bend, La.

J. J. Newton, jr., M. D., President Louisiana State Medical Society, Bastrop, La.

T. O. Brewer, M. D., chairman of the Committee of Arrangements of the 10th annual meeting of the Louisiana State Medical Society, Monroe, La.

Thomas Buffington, Baton Rouge, La.

W. R. McCreigh, M. D.

W. O. White, M. D., Vice-President Third Congressional District, Louisiana State Medical Society, Abbeville, La.

J. C. Brown, M. D., Arcadia, La.

W. E. Cushman, M. D., President Vermillion Parish Medical Society, and other members of the Louisiana State Medical Society.

